# Medical resource usage for COVID-19 evaluated using the National Database of Health Insurance Claims and Specific Health Checkups of Japan

**DOI:** 10.1371/journal.pone.0303493

**Published:** 2024-05-13

**Authors:** Keita Fukuyama, Yukiko Mori, Hiroaki Ueshima, Shiho Ito, Masaki Tanabe, Tomohiro Kuroda

**Affiliations:** 1 Division of Medical Information Technology and Administration Planning, Kyoto University Hospital, Sakyo-ku, Kyoto city, Kyoto, Japan; 2 Department of Clinical Laboratory, Mie University Hospital, Edobashi, Tsu city, Mie, Japan; 3 Department of Infection Control and Prevention, Mie University Hospital, Edobashi, Tsu city, Mie, Japan; Kyung Hee University School of Medicine, REPUBLIC OF KOREA

## Abstract

**Purpose:**

The coronavirus disease 2019 (COVID-19) pandemic exhibited several different waves threatening global health care. During this pandemic, medical resources were depleted. However, the kind of medical resources provided to each wave was not clarified. This study aimed to examine the characteristics of medical care provision at COVID-19 peaks in preparation for the next pandemic.

**Methods:**

Using medical insurance claim records in Japan, we examined the presence or absence of COVID-19 infection and the use of medical resources for all patients monthly by age group.

**Results:**

The wave around August 2021 with the Delta strain had the strongest impact on the working population in terms of hospital admission and respiratory support. For healthcare providers, this peak had the highest frequency of severely ill patients. In the subsequent wave, although the number of patients with COVID-19 remained high, they were predominantly older adults, with relatively fewer patients receiving intensive care.

**Conclusions:**

In future pandemics, we should refer to the wave around August 2021 as a situation of medical resource shortage resulting from the COVID-19 pandemic.

## Introduction

Since 2019, the coronavirus disease 2019 (COVID-19) has had a strong impact on global healthcare. Given the rapid increase in the number of COVID-19 cases, shortage in medical resources (e.g., masks and disinfectants) for infectious diseases occurred [1–6]. Multiple wards were operated exclusively for COVID-19, and other medical care services were restricted [7,8]. Considering the lack of established treatments for COVID-19 drugs, other drug such as heparin and corticosteroids were constantly used, leading to shortage [9] and causing further burden to the healthcare system. For respiratory symptoms, respiratory support therapies, such as oxygen administration, artificial respiration, and extracorporeal membrane oxygenation (ECMO) were provided. However, resources such as ventilators, ECMO equipment, intensive care units (ICUs), and medications, which were also needed by patients with non-COVID-19 diseases, were limited locally [10]. Providing these treatments to patients with COVID-19 may result in the loss of opportunities for non-COVID-19 patients who needed them. Furthermore, numerous tests were developed and implemented to diagnose COVID-19, but access to such tests was initially limited; as the number of infected people increased, test kit shortage also occurred [11,12].

Currently, the COVID-19 mortality rate is lower than it was at the beginning of the pandemic. Although caution is still necessary because of its high transmissibility, the world has gradually recovered from the COVID-19 crisis [13]. However, people periodically experience pandemics caused by unknown pathogens; thus, the next pandemic might occur at some point [14,15]. The kind of medical resources invested during each peak and the degree of impact it had on non-COVID-19 medical care remain largely unknown. In preparation for the next pandemic, the characteristics of medical care provision at multiple infection peaks should be identified.

The National Database of Health Insurance Claims and Specific Health Checkups of Japan (NDB) is a national administrative database of medical claims that has been utilized since April 2009. The NDB has covered more than 95% of electronic claims, including medication, medical procedures, examination, and, from hospitals since April 2014. This database is also widely used to verify the status of medical expenses in Japan [16–18].

In this study, we aimed to determine the impact of the novel COVID-19 pandemic on Japanese medical care provision by examining the type and amount of medical resources invested in patients with and without COVID-19 in Japan at the various peaks of the COVID-19 pandemic.

## Materials and methods

### Ethics statement

The Kyoto University Graduate School of Medicine Ethics Committee approved this study (http://www.ec.med.kyoto-u.ac.jp/) as R3620. All study procedures involving human participants conformed to the principles of the 1964 Declaration of Helsinki and any subsequent amendments or equivalent ethical standards.

Acquisition of informed consent for participation was waived in accordance with Article 16, Paragraph 2 of the Act on Securing Medical Care for the Elderly in Japan.

### Dataset preparations

In Japan, all citizens and long-term residents can avail themselves of a universal health insurance through the National Health Insurance System. After being anonymized, claims for medicines and services available in almost all national health insurances are collected and stored in the NDB. Researchers can directly analyze the data stored in the NDB in the Onsite Research Center, a specified analysis environment with the prescribed application and permission.

This retrospective observational study was conducted using NDB data recorded from January 2020, when the severe acute respiratory syndrome coronavirus 2 (SARS-CoV-2) was first detected in Japan, to December 2022. We extracted the medical records of all citizens during said period, imported them into the PostgreSQL database existing in the Onsite Research Center, and then analyzed them. We did not have access to information that could identify individual participants either during or after data collection.

### Definition of patients

We used ID1n, a hash value generated from the insurer’s ID, beneficiary’s ID, birth date, and sex, as a patient identifier among patient IDs in NDB. NDB has a rule that IDs, such as insured person’s ID cannot be directly used. In NDB, the ID1n changes even for a single patient, mainly because of a change of insurer; therefore, following up a single patient over a long period and investigating the utilization of medical resources can be difficult. Hence, we aggregated the data on a person-month basis.

We defined the code corresponding to acute-phase COVID-19 infection from the disease name master used for making claims (S2 Table). Patients assigned with a COVID-19 disease code without a suspicion flag were defined as patients with COVID-19 and then compared with those without a COVID-19 disease name in each month. Patients were stratified by age as follows: young (<15 years), adult (15–64 years), and older (>64 years).

To verify the validity of the abovementioned definitions, we compared the number of patients defined in this study with the number of newly infected patients in the open data provided by HER-SYS, information acquisition, and management system for new COVID-19 cases provided by the Ministry of Health, Labor and Welfare (https://covid19.mhlw.go.jp/extensions/public/index.html). In Japan, all cases of COVID-19 must be reported until March 2023, and data for all cases are registered in HER-SYS [19]. During this time, all diagnoses of COVID-19 in Japan were classified by the following four patterns:

- Patient (confirmed case): A patient suspected of infection and with a positive SARS-CoV-2 test result;
- Asymptomatic pathogen carrier: A person with no symptoms but a positive SARS-CoV-2 test result;
- Suspected patient: A patient suspected to be infected and with a high clinical probability of requiring hospitalization; and
- Corpse of a person who died of a (suspected) infectious disease: A person who died of COVID-19 or is suspected to have died of COVID-19.

PCR or antigen test was not mandatory for categories iii and iv at these registrations. As secondary insurance benefits for these patients are not registered in the NDB, they are not included in this analysis.

### Medical resource usage

We examined the number of patients with or without COVID-19 admitted to general wards, ICUs, and psychiatric wards. We extracted claim codes corresponding to the use of oxygen administration, artificial respiration, and ECMO as respiratory support and counted the number of patients who received these medical treatments. Furthermore, the use of sedatives, corticosteroids, and anticoagulants, which were feared to be depleted during the pandemic, was assessed. We then counted the total amount of drugs used in vials, ampoules, tablets, capsules, and more. As it is not possible to obtain certain information, such as body size or disease severity, from receipt information, the total amount of drugs administered to each patient is calculated without dosage compensation or adjustments based on any standard or international treatment protocols.

Regarding the implementation status of tests for diagnosing COVID-19, we classified the codes according to whether the detection target was nucleic acid or antigen, and whether the test was outsourced or not. Subsequently, we counted the number of tests performed. To validate the total medical expenses invested in patients with COVID-19, we stratified and validated the total insurance claims and public subsidies by age and COVID-19 status. Dental claims were excluded from this tabulation. Infectious disease experts determined the codes used for tabulation. We calculated the sum of claims for insurance claims and public subsidies, stratified by patients’ age and presence of COVID-19, followed by verification.

### Relationship with other pandemic respiratory viral infections

To ascertain the control status of other respiratory viral infections under COVID-19 control, we visualized the trends of the number of records of influenza virus and respiratory syncytial virus (RSV) infections, along with the trends of COVID-19 infections. S2 Table lists these codes.

### Analysis environment

For our research, we accessed data from January 24 to July 24, 2023. The analysis was conducted in a prebuilt environment using R [20] (version 4.1.2)/RStudio [21] (version 2021.09.01 Build 372) on Ubuntu 20.05.5 LTS at a closed network. The packages and versions used at the stages of extraction and aggregation are listed in the html text on GitHub. The main packages used were DBI [22] and RpostgreSQL [23] to connect to the database, tidyverse for data handling, and data.table [24] and openxlsx [25] to read and output data.

Furthermore, the local database was built using PostgreSQL (version 14.6), and the NDB dataset was extracted from Redshift (version 1.0.50468) on Amazon Web Service.

## Results

### Patient distribution

**Fig 1:**
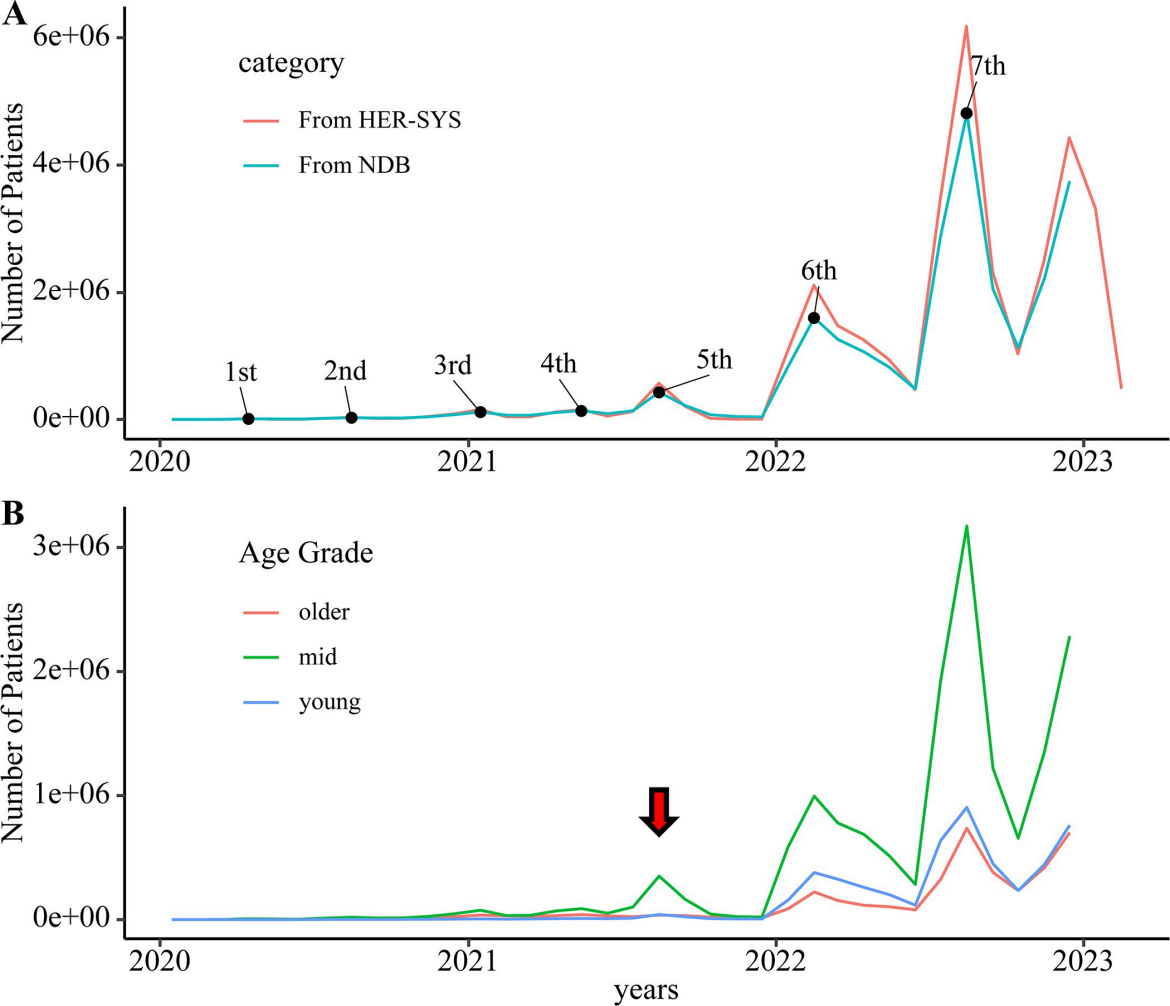
Time course of patients with COVID-19. A: The line graph shows the number of patients with COVID-19 recorded and extracted from NDB and HER-SYS. In the graph, 2e+06 indicates 2,000,000. The black dots represent the peak of each wave. B: Age grade of patients with COVID-19. The red arrow indicates the 5th wave. During this wave, most of the patients with COVID-19 were adults, while older adults and young individuals infected with the disease were few. Please note that the vertical axis scales of the two graphs are different.

From January 2020 to December 2022, 160 million IDs were collected, of which 20 million had at least one COVID-19 diagnosis. S1 Fig shows the distribution of patients’ identifier and COVID-19 infection status. The trend in number of new patients in HER-SYS was consistent with the that of person-months of COVID-19 infection on the NDB (Fig 1A). S2 Fig shows a graph evaluated on a logarithmic axis together with patients without a COVID-19 disease name. We identified seven separate infection waves and peaks from the data available in the NDB, according to the trends in the number of infections. We tagged these peaks with numbers 1 to 7. S1 Table shows the month of each peak.

Regarding the trends of the number of patients with COVID-19 by age group, we noted that many adults, who mainly constitute the working population, were infected in the 5th wave in August 2021, when the Delta strain became a burden. At the subsequent peak, the number of older and young patients increased (Fig 1B).

### Use of medical resources

#### Bed occupancy

**Fig 2:**
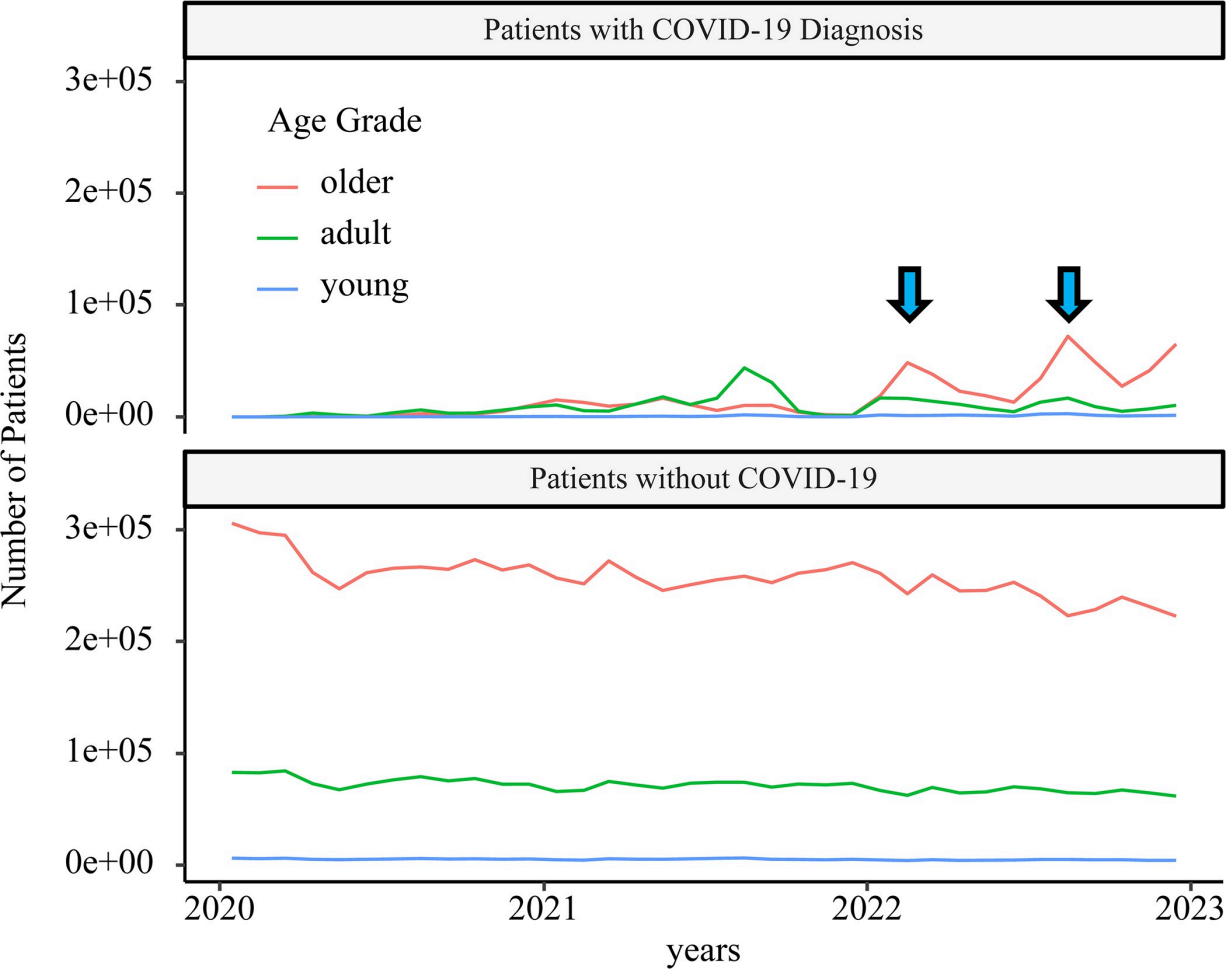
Time course of general ward admissions. The blue arrows indicate the 6^th^ and 7^th^ waves. During these waves, general ward admissions of patients without COVID-19 decreased.

The number of admissions to the general wards for patients with COVID-19 showed a clear peak during the 5^th^ wave, but in the 6^th^ and 7^th^ waves, the number mildly increased (Fig 2). In the 5^th^ wave, many adults were hospitalized, but in the following peak, most of the hospitalized patients were older adults. In the 6^th^ and 7^th^ waves, the number of admissions of patients without COVID-19 decreased according to the peak.

**Fig 3:**
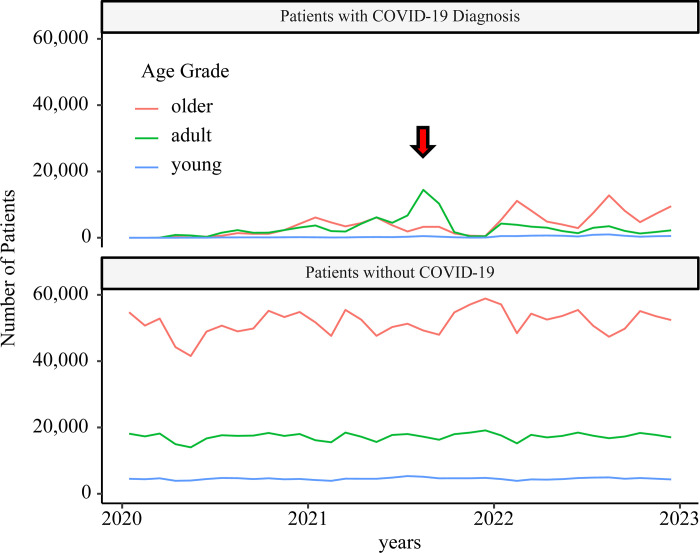
Time course of intensive care unit (ICU) usage. The red arrow indicates the 5^th^ wave. Numerous adult patients with COVID-19 were admitted to the ICU during this wave. In the subsequent waves, ICU usage decreased.

Furthermore, most of the patients with COVID-19 admitted to the ICU at the 5^th^ peak were adults. In the 6^th^ and 7^th^ peaks, majority of patients with the disease were older adults; meanwhile, the number of patients without COVID-19 did not decrease (Fig 3). From the 6^th^ and 7^th^ waves, patients with COVID-19 were allowed to be admitted to the psychiatric wards. At the same time, the number of patients without COVID-19 admitted to the general wards decreased (S3 Fig).

#### Medical commodities

**Fig 4:**
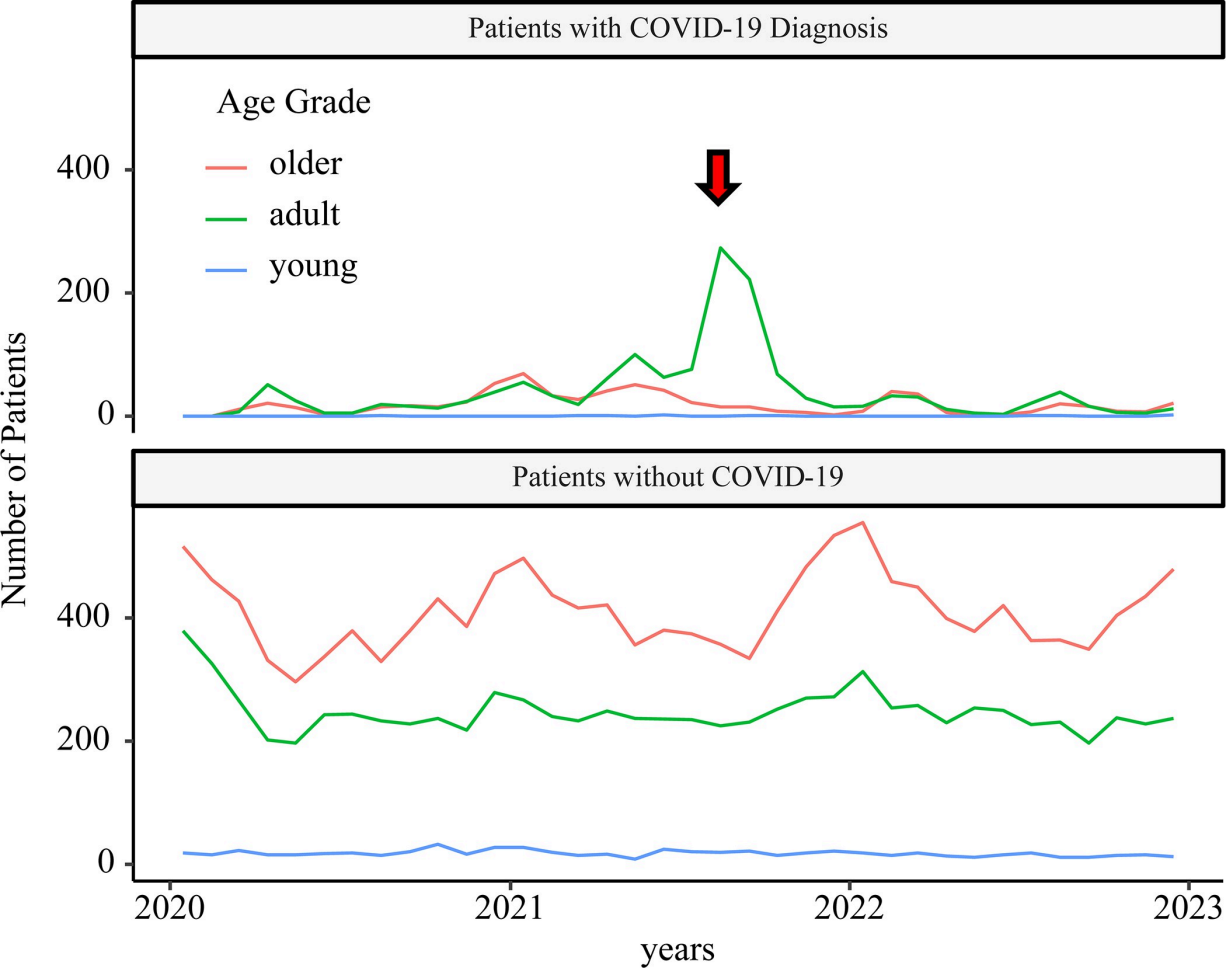
Time course of ECMO usage. The red arrow indicates the 5^th^ wave. During this wave, ECMO was mostly administered to adult patients with COVID-19. In later waves, very few people with the disease were treated with ECMO.

ECMO was mostly used by adult patients with COVID-19. However, the downward trend of ECMO use in patients without COVID-19 was unclear, even in the 5^th^ wave (Fig 4). After the 6^th^ and 7^th^ waves, although the number of admitted patients was high, ECMO use was minimal. S4 and S5 Figs show the time course of artificial respiration and oxygen administration, respectively.

**Fig 5:**
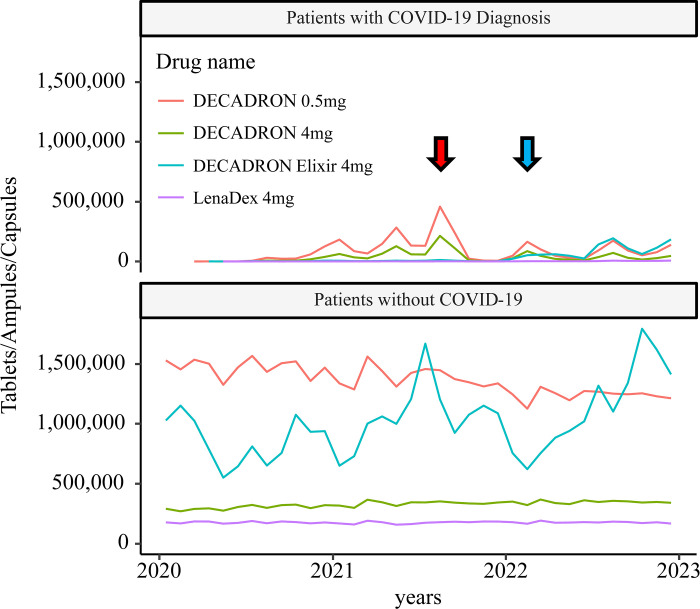
Time course of oral corticosteroid usage. The red and blue arrows indicate the 5^th^ and 6^th^ waves. In the 5^th^ wave, when many corticosteroids were prescribed, the decrease in corticosteroid prescriptions for patients without COVID-19 was unclear. However, corticosteroid prescription for patients with COVID-19 decreased markedly since the 6^th^ wave.

Drug use differed between the 5^th^ and 6^th^ waves. The use of sedatives, anticoagulants, and corticosteroids declined relatively in the 6^th^ wave compared with that in the 5^th^ wave. Fig 5 shows the time course of oral corticosteroid use. Overall, the decrease in drug use among patients without COVID-19 in response to drug administration among those with COVID-19 at each peak was unclear. Changes in other drug categories are shown in S6–S11 Figs.

**Fig 6:**
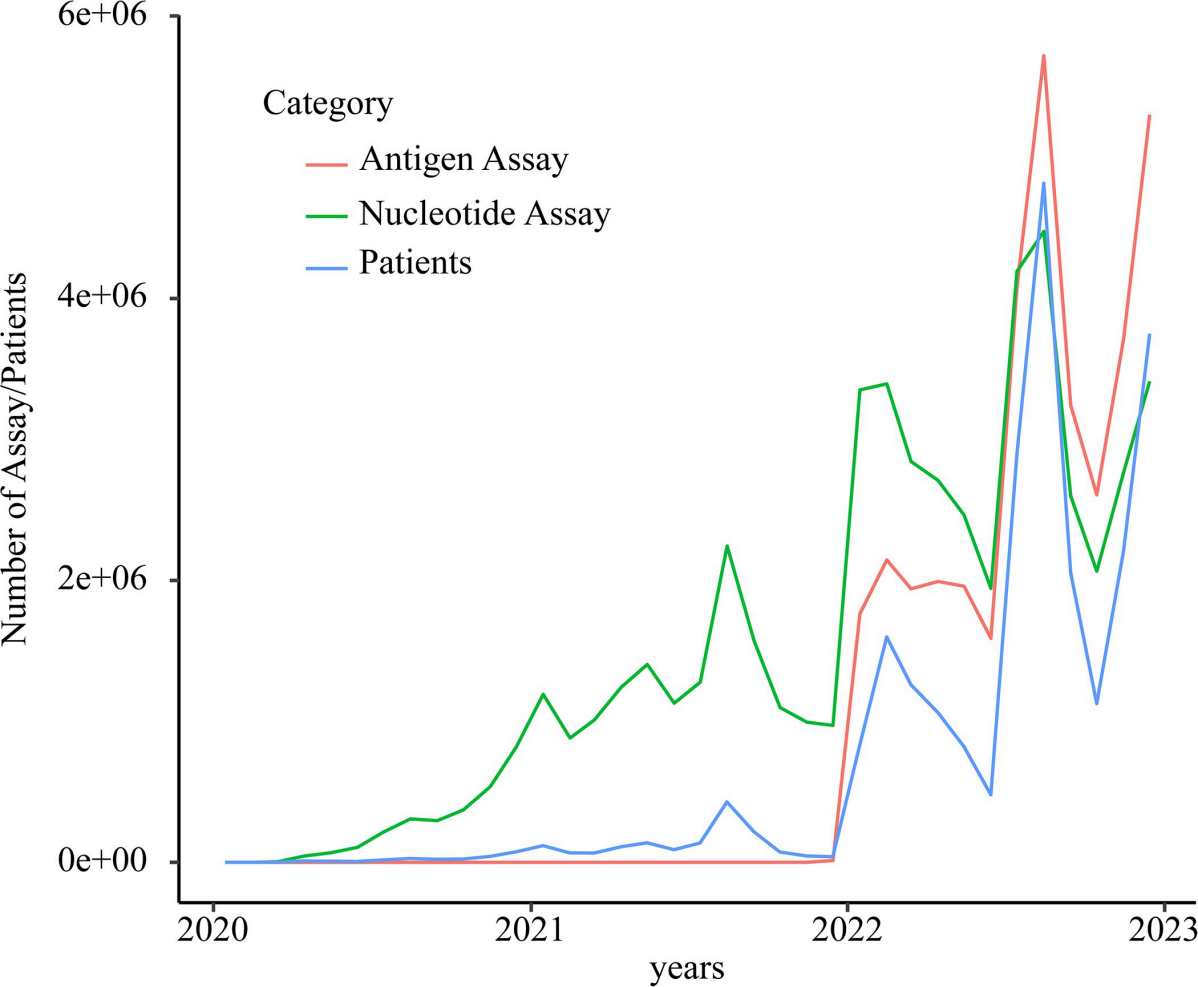
Time course of assay usage. The primary method for diagnosing the virus was through nucleic acid detection until the 5^th^ wave. However, as the 6^th^ wave emerged, the use of antigen tests significantly surged, leading to their widespread implementation in large quantities.

The main test for diagnosing COVID-19 was nucleic acid detection until the 5th wave (Fig 6). Since the 6th wave, antigen tests had become widespread and been performed in large numbers. The sales and popularization of easy-to-use antigen tests occurred 2 years after the arrival of the new coronavirus in Japan.

#### Financial cost to health insurance

**Fig 7:**
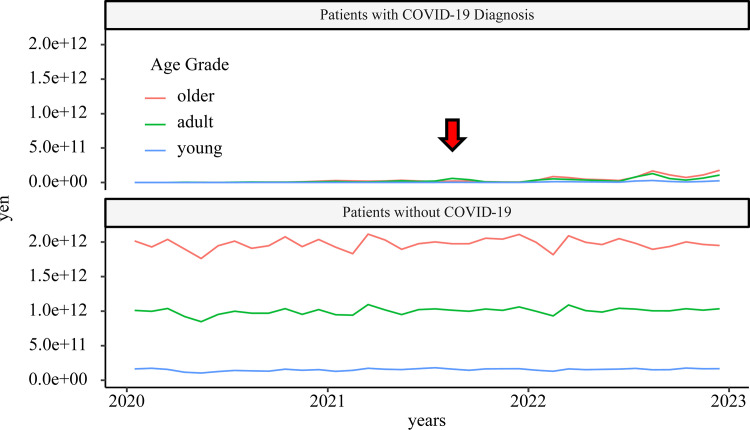
Time course of total medical claims. The red arrow indicates the 5^th^ wave. During this wave, the percentage of medical care invested in patients with COVID-19 was smaller than that in those without COVID-19.

Medical expenses for older adults were overwhelmingly large in Japan. For patients with COVID-19, the medical expenses against the overall medical expenses became clear during the 6th wave. In the 5th wave, medical resources were highly consumed by intensive care for patients with COVID-19, but the proportion of the total medical expenses in Japan was not large (Fig 7). S12 Fig shows changes in medical expenses converted into US dollars at the average exchange rate for the month.

### Relationship with other respiratory viral infections

**Fig 8:**
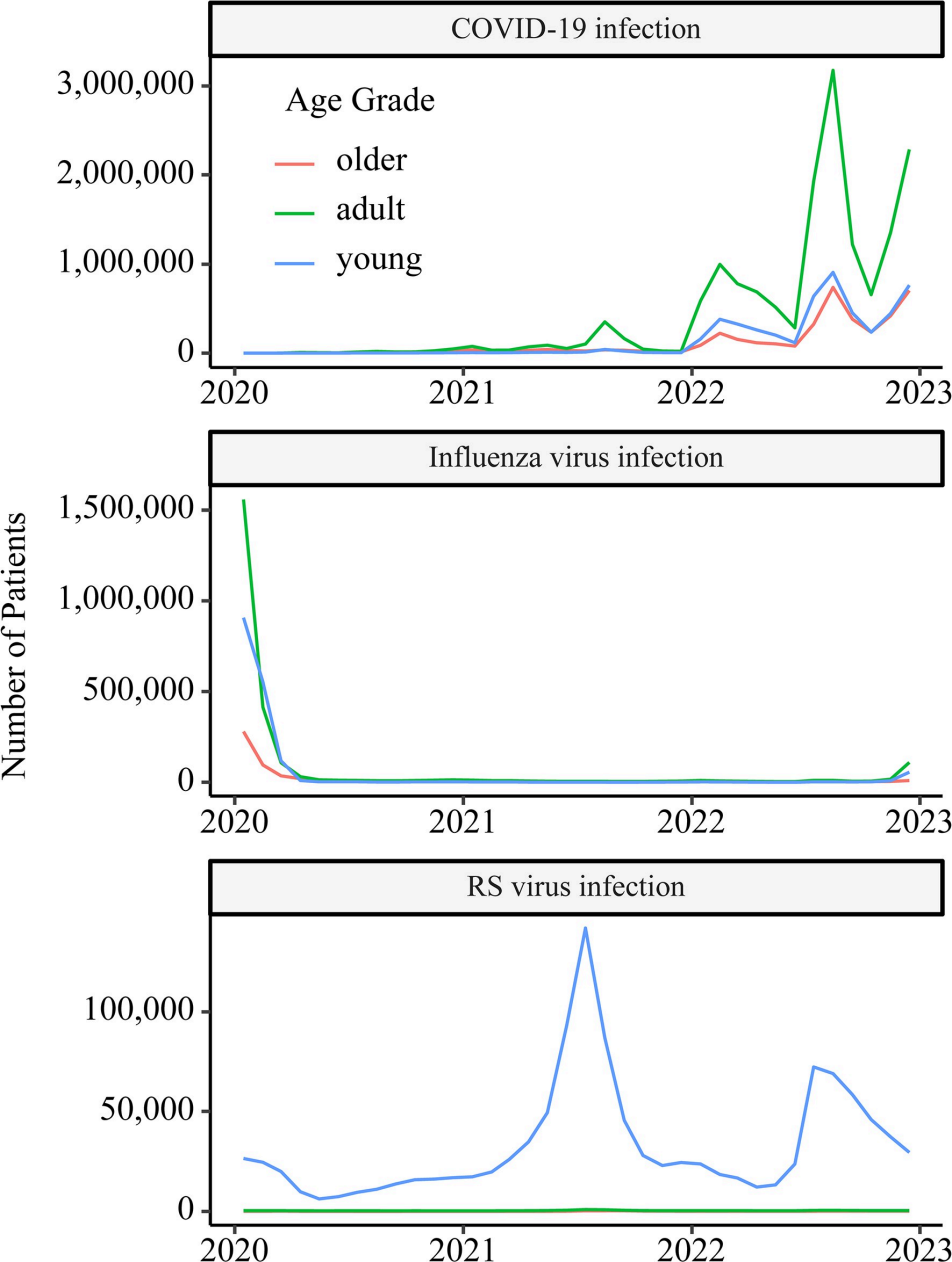
Time course of three respiratory viral infections. The COVID-19 crisis has led to a significant reduction in the number of influenza cases, with no apparent peak observed. Conversely, RSV infections notably surged amid the COVID-19 crisis.

The number of patients with influenza drastically decreased during the COVID-19 crisis, with no clear peak comparable to usual years (Fig 8). Conversely, the number of patients with RSV was high, indicating the difficulty of controlling the spread of this infection.

## Discussion

We identified COVID-19 trends from the NDB and the corresponding patient resource usage. Fig 1A shows that the number of patients with COVID-19 in the NDB was similar to in the number of reports in the HER-SYS, allowing estimation of the infection status using receipt information. During the 5^th^ wave around August 2021—when Delta strain became a problem—hospital admissions among the working population and an increase in the use of oxygen therapy were observed, indicating a significant impact on this group. At the peak after the omicron strain became a problem, few hospitalizations and respiratory support were noted among the working population.

Multiple factors other than the virus strains may be responsible for large differences in patient distribution and medical resource usage between the 5th, 6th, and 7th peaks. Regarding COVID-19 vaccines, the Pfizer vaccine was approved in Japan for manufacturing and sale on February 14, 2021, and vaccination began on February 17, 2021. Vaccination with Takeda/Moderna’s vaccine began on May 24, 2021, while vaccination with the AstraZeneca vaccine began on August 2, 2021. Initially, vaccinations were administered to healthcare workers and the elderly; however, on June 17, 2021, people aged 18–64 were considered eligible for vaccination. It is estimated that ≥50% of the population aged ≥65 completed two vaccinations on July 13, 2021, while ≥50% of the population aged <65 completed two vaccinations on October 14, 2021 [26], which was after the fifth peak. The Tokyo Olympics took place before the fifth peak, and may have encouraged movement by young people before vaccination. Although the omicron strain was highly infectious, the rate of severe illness is reportedly lower than that of other strains [27,28]. While some vaccines have low efficacy at preventing infection by the omicron strain, vaccination is known to prevent serious illness after infection [29]. These factors may explain the differences between wave 5 and waves 6 and 7.

As noted by Kwon et al., there are large differences in COVID-19 vaccination rates between countries [30]. Vaccination rates are low in economically developing countries, and it may be challenging to provide the generous levels of respiratory support provided in Japan for severely ill unvaccinated patients. In the case of a future pandemic, careful discussions will be needed to determine what groups should be vaccinated in what order considering the time course in Japan.

Our results provide several implications for future pandemics. For the 6th and 7th waves, when the number of patients increased, there was a decrease in claims for patients without COVID-19 who were admitted to general and psychiatric wards, suggesting that COVID-19 care was putting pressure on non-COVID-19 care. This may be due to the fact that COVID-19 patients were also being admitted to general and psychiatric beds at this time. These phenomena were observed from around the time the monthly number of patients exceeded 1 million. This number, 1 million COVID-19 patients at one month, could be an indicator that the pandemic will begin to overwhelm medical care for other diseases. During the time period of this study, there was a marked drop in the number of influenza cases. This suggests that if the infectivity of influenza during a pandemic is the same as it is today, the same infection prevention measures as those used against COVID-19 may be able to strongly control influenza transmission. In contrast, the RSV was widespread, especially among young people, and the COVID-19 countermeasures in Japan which did not recommend the use of face masks by children aged <2 years, were insufficiently effective for RSV infection [31].

As for respiratory support, although the use of ventilators and ECMO increased during the 5th wave, the impact on non-COVID-19 patients was minimal, and the limits of simultaneous use of ECMO and ventilation in Japan are unclear from the present verification. As for medication, around April 2021, along with an increase in patients with severe disease the use of propofol increased dramatically, resulting in supply shortage and a notice requesting the drugs proper use. In August 2021, a shortage of dexamethasone occurred, and a notice was issued requesting that steroids not be used for patients with mild to moderate COVID-19 infection. This analysis did not find any significant impact on the peak of COVID-19 infection, such as restrictions on the amount of the relevant drug prescribed in treating non-COVID-19 patients. Although it was clear that the peak of COVID-19 infection would place a heavy burden on the pharmaceutical supplies, the proper use of medications did not lead the depletion of these resources for non-COVID-19 patients in Japan.

This study has several limitations. The NDB only covers medical practices at medical institutions; thus, it cannot verify self-funded tests and treatments that are not covered by health insurance. In addition, the diagnosis information of COVID-19 depends on the disease naming in insurance medical treatment; hence, it is not necessarily based on the virological diagnosis and asymptomatic infected people, who accounted for 0.33% of the population at the fifth peak [32], are not registered as COVID-19 infected patients unless tested for some reason. Although prescriptions of Mornupiravir were seen among those without COVID-19 disease, and there was a tendency to count more patients than HER-SYS in non-peak months when the number of patients declines, we confirmed that the disease name definitions used this time corresponded to the trend of the epidemic from the HER-SYS. Considering that HER-SYS will no longer register all cases after May 2023, we consider that the NDB is one of the promising approaches to evaluate the disease activity of COVID-19 in the population because this method is similar to comprehensive surveillance in clinical practice in Japan [33].

Several studies have evaluated COVID-19 pandemic in Japan using NDB, including changes in characteristics and outcomes of COVID-19 patients from the early pandemic to the Delta variant epidemic [34], and changes in the number of outpatient visits during the COVID-19 pandemic [35]. However, this is the first study to evaluate COVID-19 impact using the NDB in Japan from the perspective of medical burden, including the phase of the omicron variant epidemic.

In conclusion, this study reveals that amongst all the COVID-19 infection peaks, the 5^th^ wave caused by the Delta strain had the strongest impact on the working population, and responding to severe cases is necessary. For future pandemics, we should refer to the 5^th^ wave as a situation of severe infectious diseases with a high mortality rate. In addition, validation of receipt data is a promising method for assessing pandemics.

## Supporting information

S1 Fig Distribution of patients’ identifiers by age and COVID-19 infectious status. Bar plots show the patients’ age and whether the disease name of acute COVID-19 was assigned at least once for all 160 million IDs for which claims were recorded over the past 3 years.
(TIF)

S2 Fig Time course of patients with COVID-19 who had HER-SYS data in the log scale. The line graph shows the number of patients with and without COVID-19 recorded and extracted from NDB and HER-SYS. We have added 1 to the number of patients for logarithmic display.
(TIF)

S3 Fig Time course of psychiatric ward admissions. The blue arrows indicate the 6^th^ and 7^th^ waves. During these waves, psychiatric ward admissions of patients without COVID-19 decreased.
(TIF)

S4 Fig Time course of artificial respiration. In Japan, older adults tended to use artificial respiration during winter. The declining trend of mechanical ventilation among patients without COVID-19 in response to artificial respiration increase among patients with COVID-19 was unclear.
(TIF)

S5 Fig Time course of oxygen administration. The downward trend of oxygen administration in patients without COVID-19 in response to oxygen administration increase in patients with COVID-19 was unclear.
(TIF)

S6 Fig Time course of low-molecular-weight heparin. The specifications of low-molecular-weight heparin over time, stratified by the presence or absence of COVID-19 infection.
(TIF)

S7 Fig Time course of unfractionated heparin. Usage status of unfractionated heparin over time, stratified by the presence or absence of COVID-19 infection.
(TIF)

S8 Fig Time course of midazolam. Usage status of midazolam over time, stratified by the presence or absence of COVID-19 infection.
(TIF)

S9 Fig Time course of propofol. Usage status of propofol over time, stratified by the presence or absence of COVID-19 infection.
(TIF)

S10 Fig Time course of corticosteroid injection. Usage status of corticosteroid injection over time, stratified by the presence or absence of COVID-19 infection.
(TIF)

S11 Fig Time course of antiviral agents. Usage status of antiviral agents for COVID-19 over time, stratified by the presence or absence of COVID-19 infection.
(TIF)

S12 Fig Time course of total medical claims in US dollars. The red arrow indicates the 5^th^ wave. During this wave, the percentage of medical care invested in patients with COVID-19 was smaller than that in those without COVID-19. Although medical expenses appeared to have declined, such decline was caused by the impact of the yen’s depreciation.
(TIF)

S1 Table Definition of each wave. (XLSX)

S2 Table Codes used in this study. (XLSX)

S1 File Detailed methods used in this study. (DOCX)
