# Supplementary figures and images for: Medical resource usage for COVID-19 evaluated using the National Database of Health Insurance Claims and Specific Health Checkups of Japan
Source: PLoS One. 2024 May 13;19(5):e0303493. doi: 10.1371/journal.pone.0303493 (PMC11090316; doi:10.1371/journal.pone.0303493)

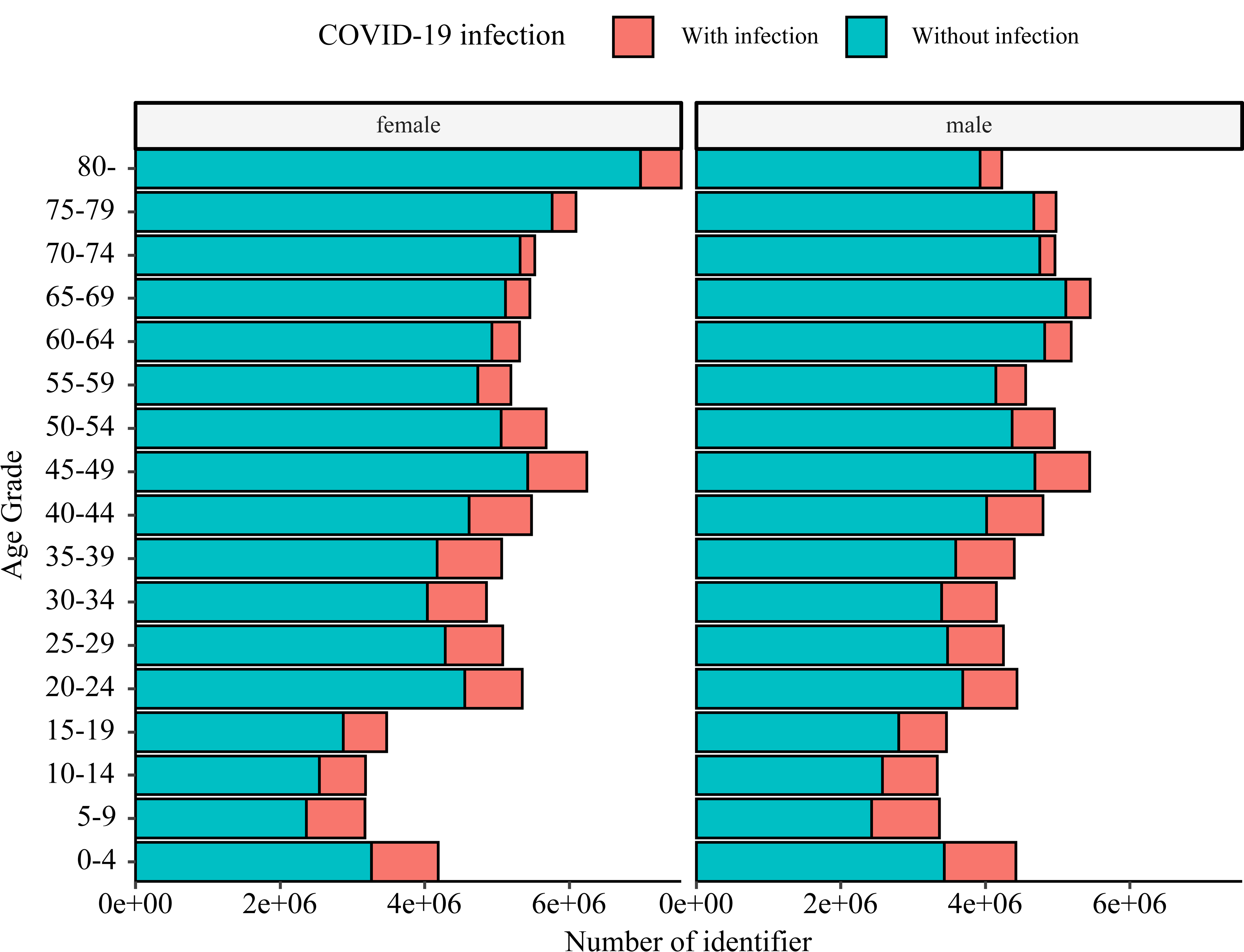

Supplement: S1 Fig — Bar plots show the patients’ age and whether the disease name of acute COVID-19 was assigned at least once for all 160 million IDs for which claims were recorded over the past 3 years. (TIF) [file pone.0303493.s001.tif]

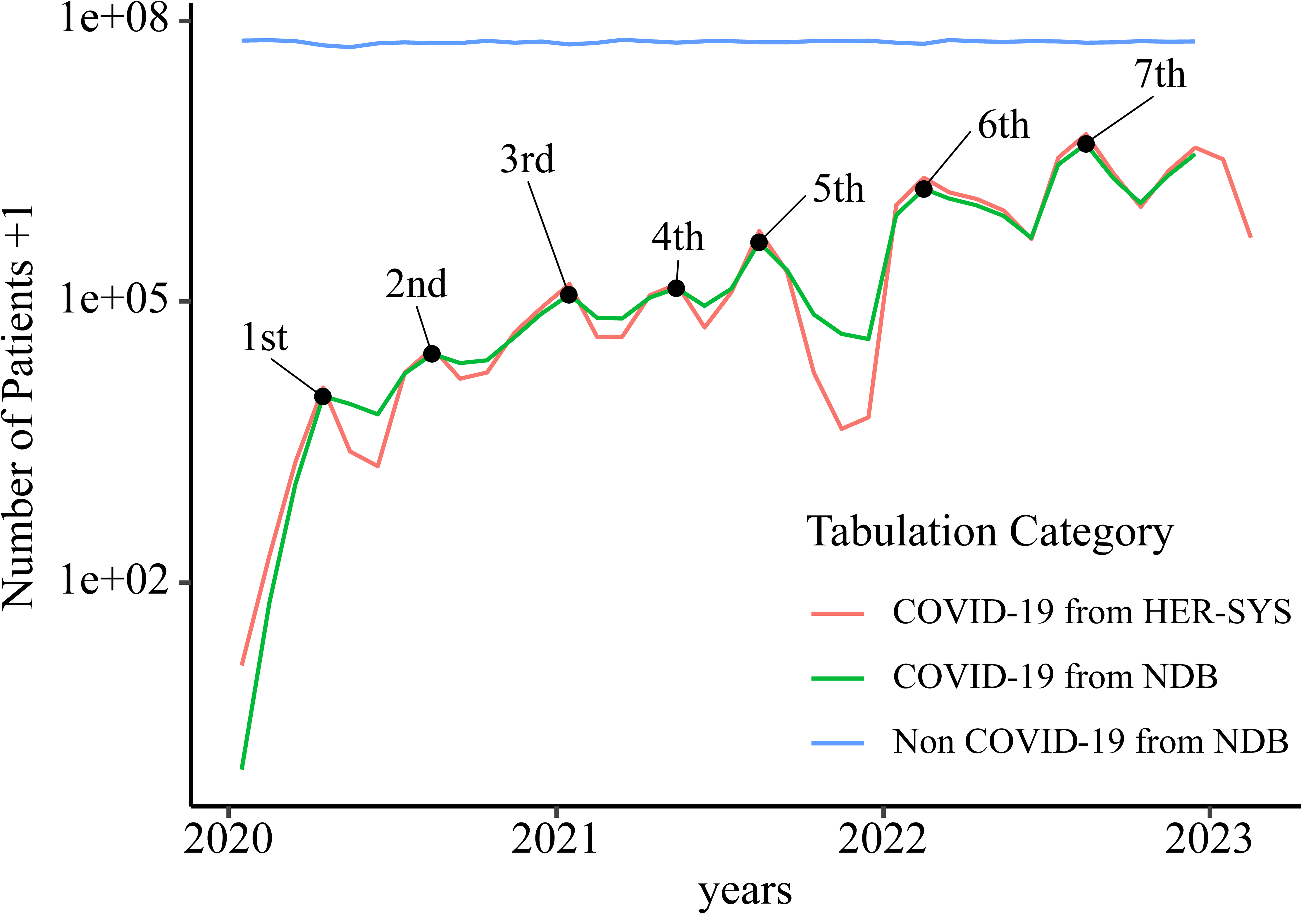

Supplement: S2 Fig — The line graph shows the number of patients with and without COVID-19 recorded and extracted from NDB and HER-SYS. We have added 1 to the number of patients for logarithmic display. (TIF) [file pone.0303493.s002.tif]

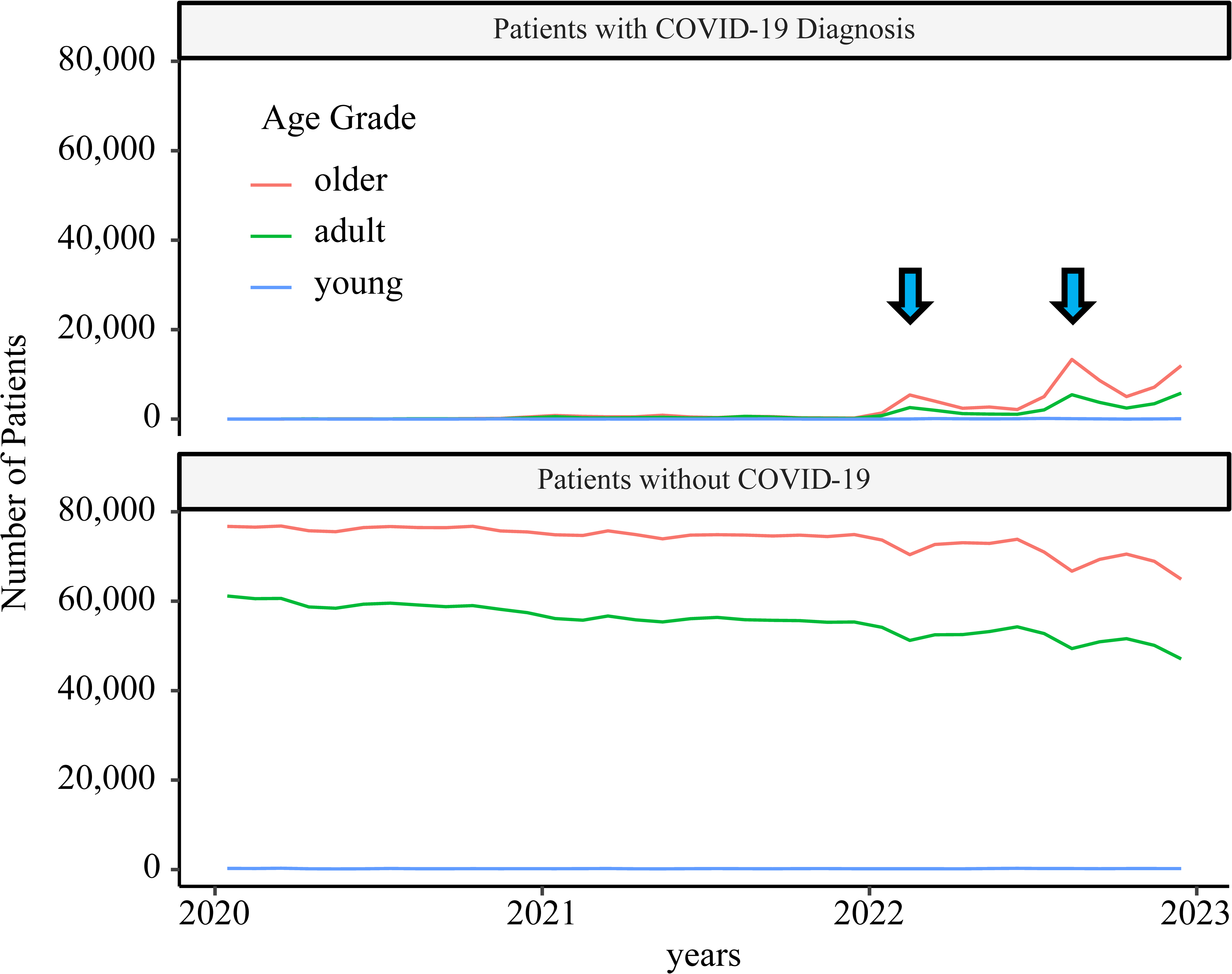

Supplement: S3 Fig — The blue arrows indicate the 6th and 7th waves. During these waves, psychiatric ward admissions of patients without COVID-19 decreased. (TIF) [file pone.0303493.s003.tif]

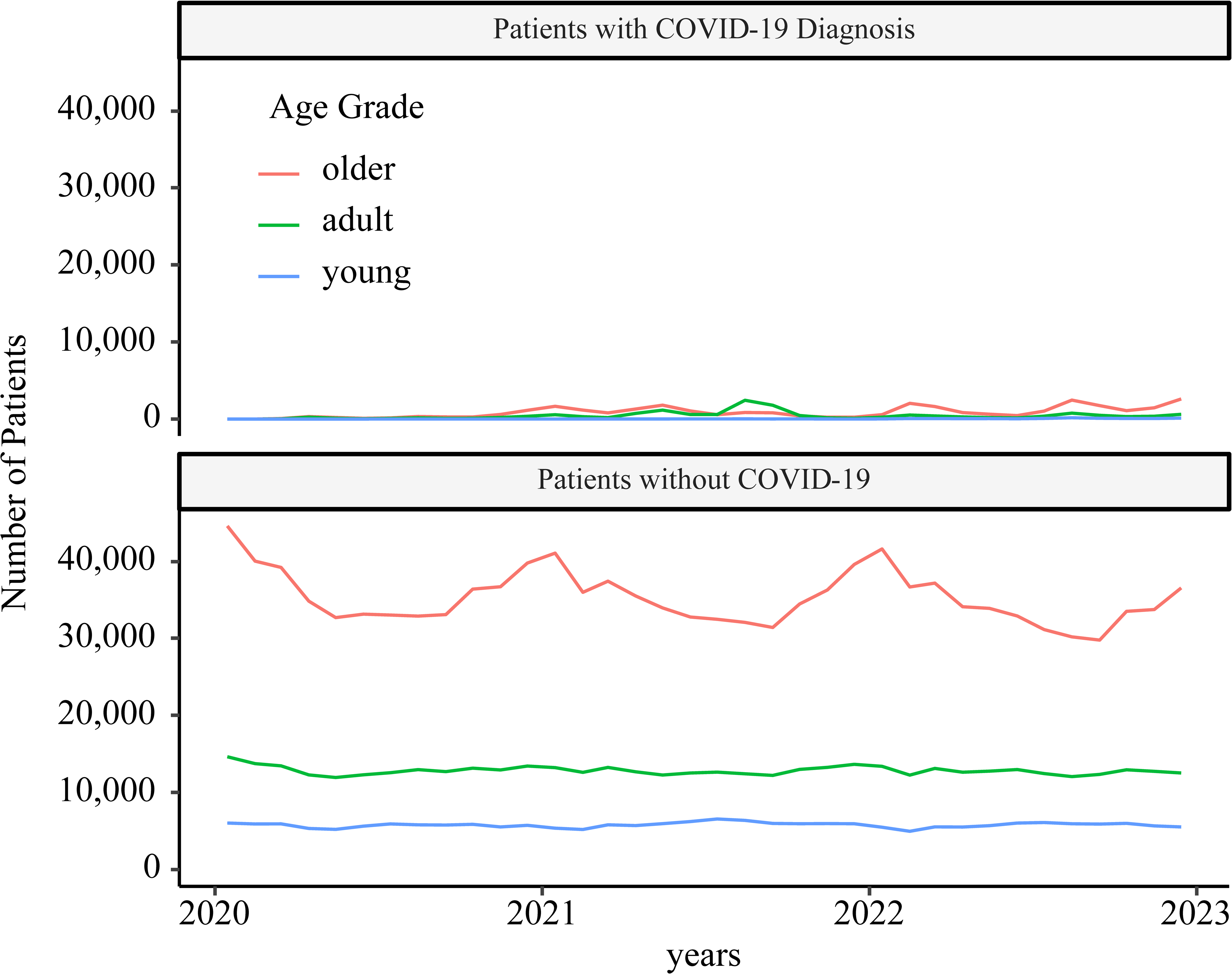

Supplement: S4 Fig — In Japan, older adults tended to use artificial respiration during winter. The declining trend of mechanical ventilation among patients without COVID-19 in response to artificial respiration increase among patients with COVID-19 was unclear. (TIF) [file pone.0303493.s004.tif]

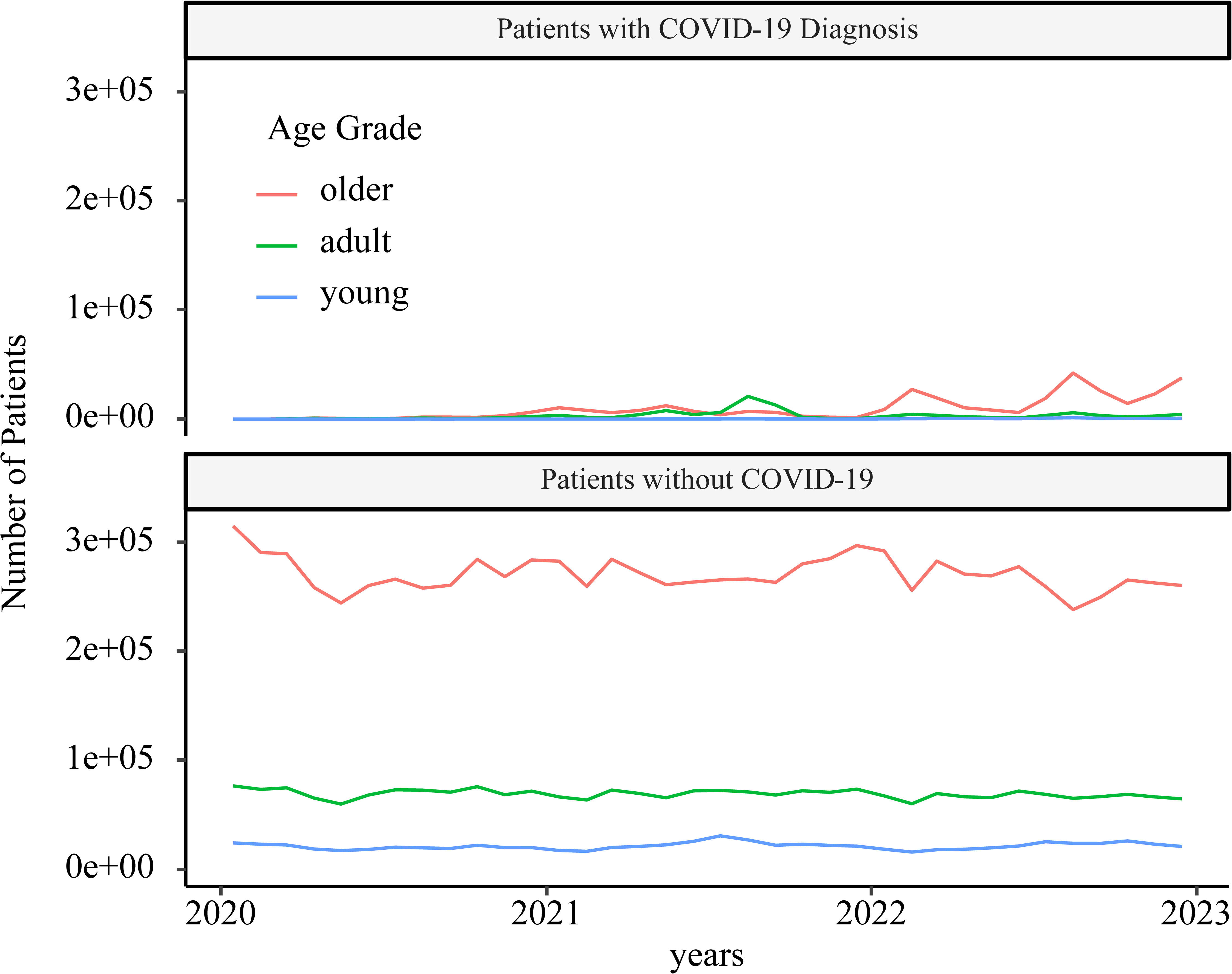

Supplement: S5 Fig — The downward trend of oxygen administration in patients without COVID-19 in response to oxygen administration increase in patients with COVID-19 was unclear. (TIF) [file pone.0303493.s005.tif]

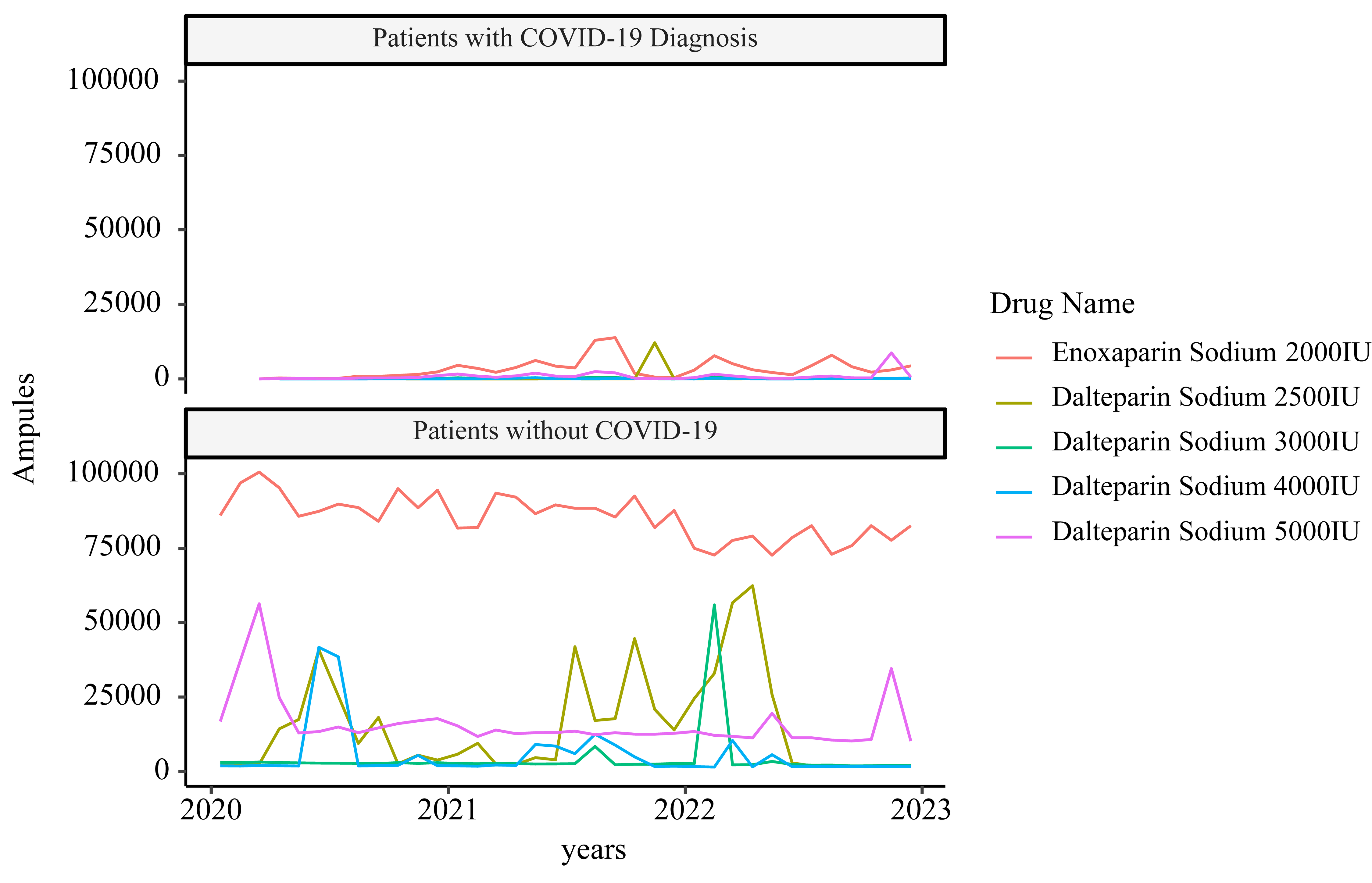

Supplement: S6 Fig — The specifications of low-molecular-weight heparin over time, stratified by the presence or absence of COVID-19 infection. (TIF) [file pone.0303493.s006.tif]

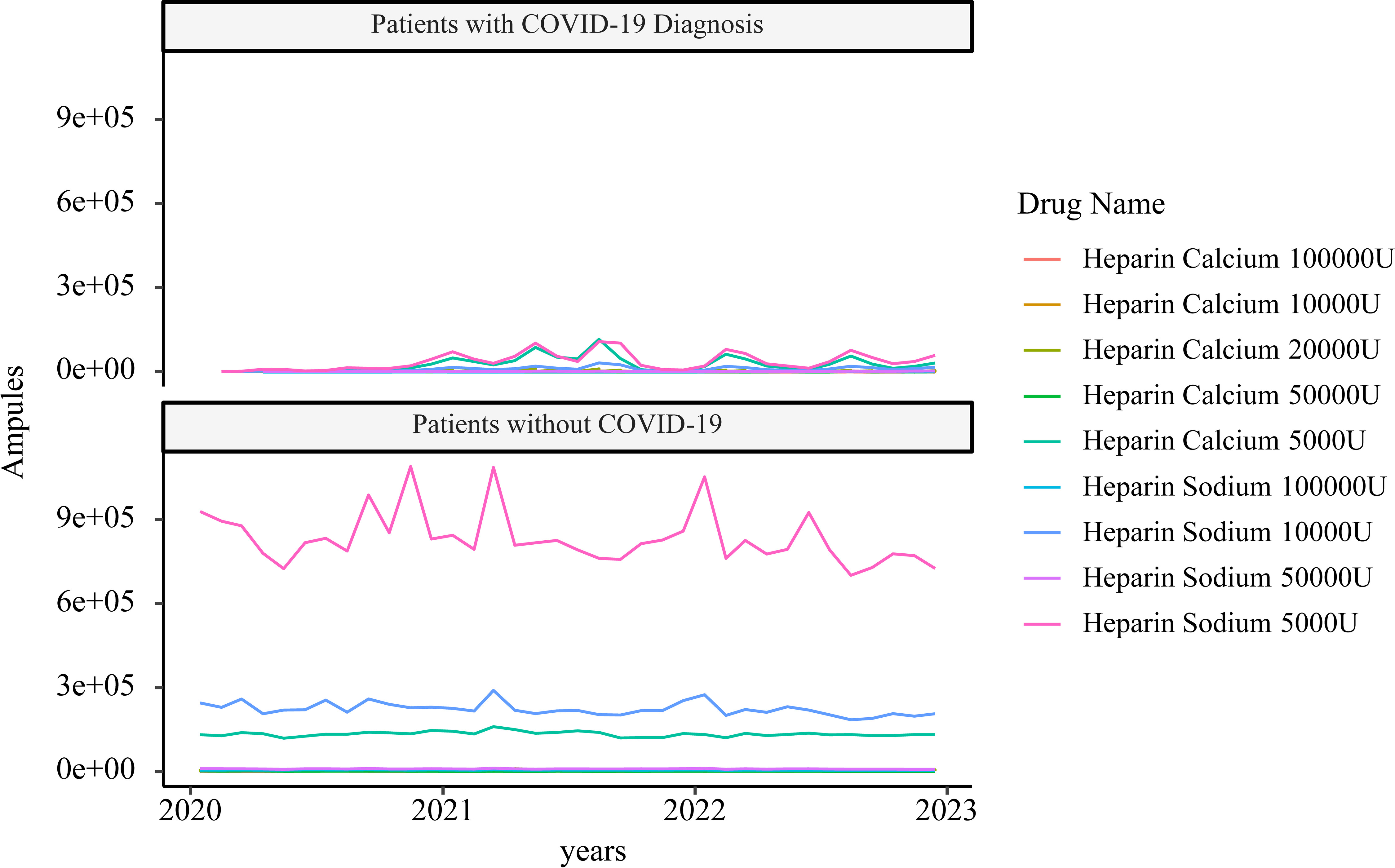

Supplement: S7 Fig — Usage status of unfractionated heparin over time, stratified by the presence or absence of COVID-19 infection. (TIF) [file pone.0303493.s007.tif]

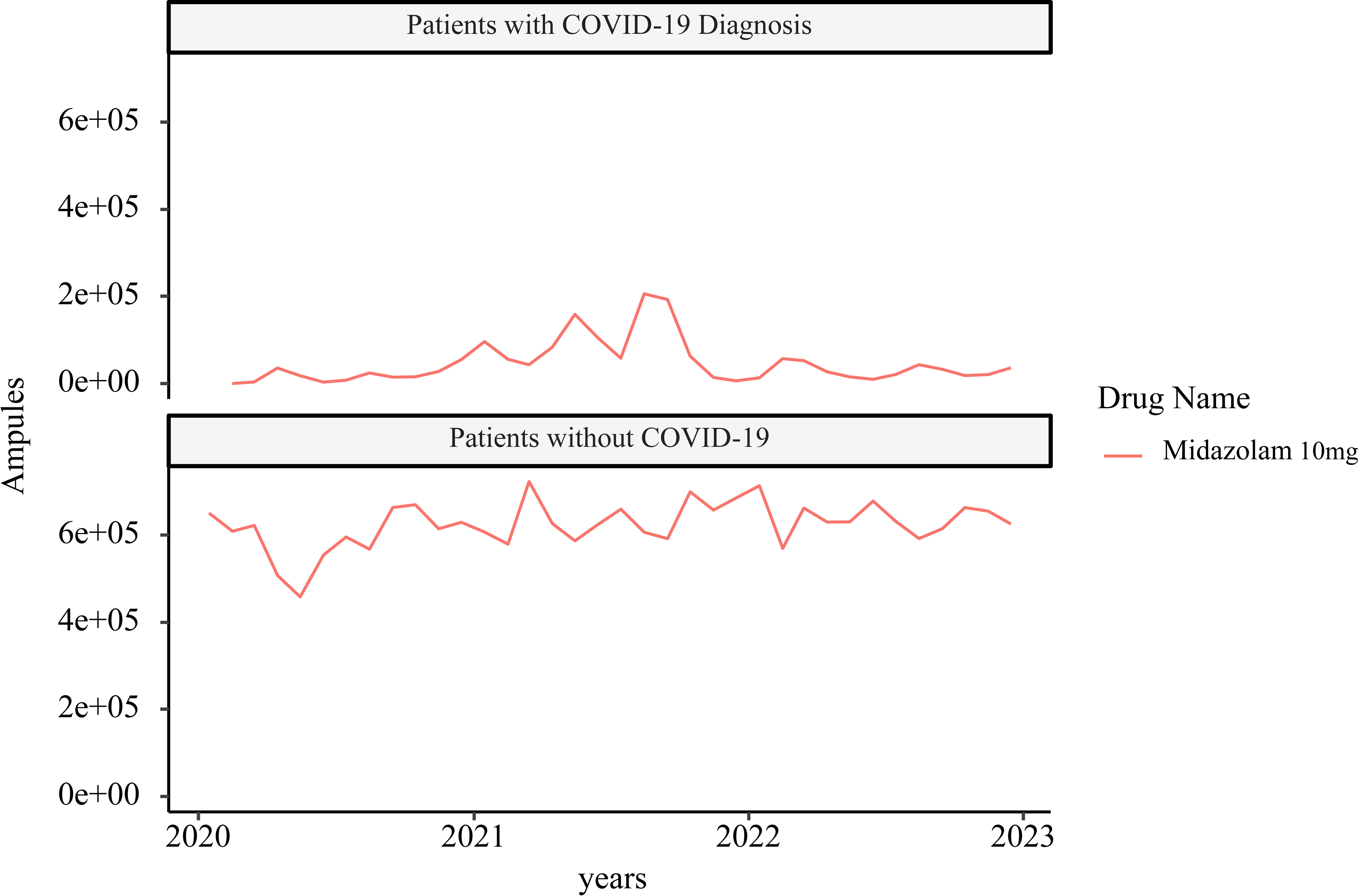

Supplement: S8 Fig — Usage status of midazolam over time, stratified by the presence or absence of COVID-19 infection. (TIF) [file pone.0303493.s008.tif]

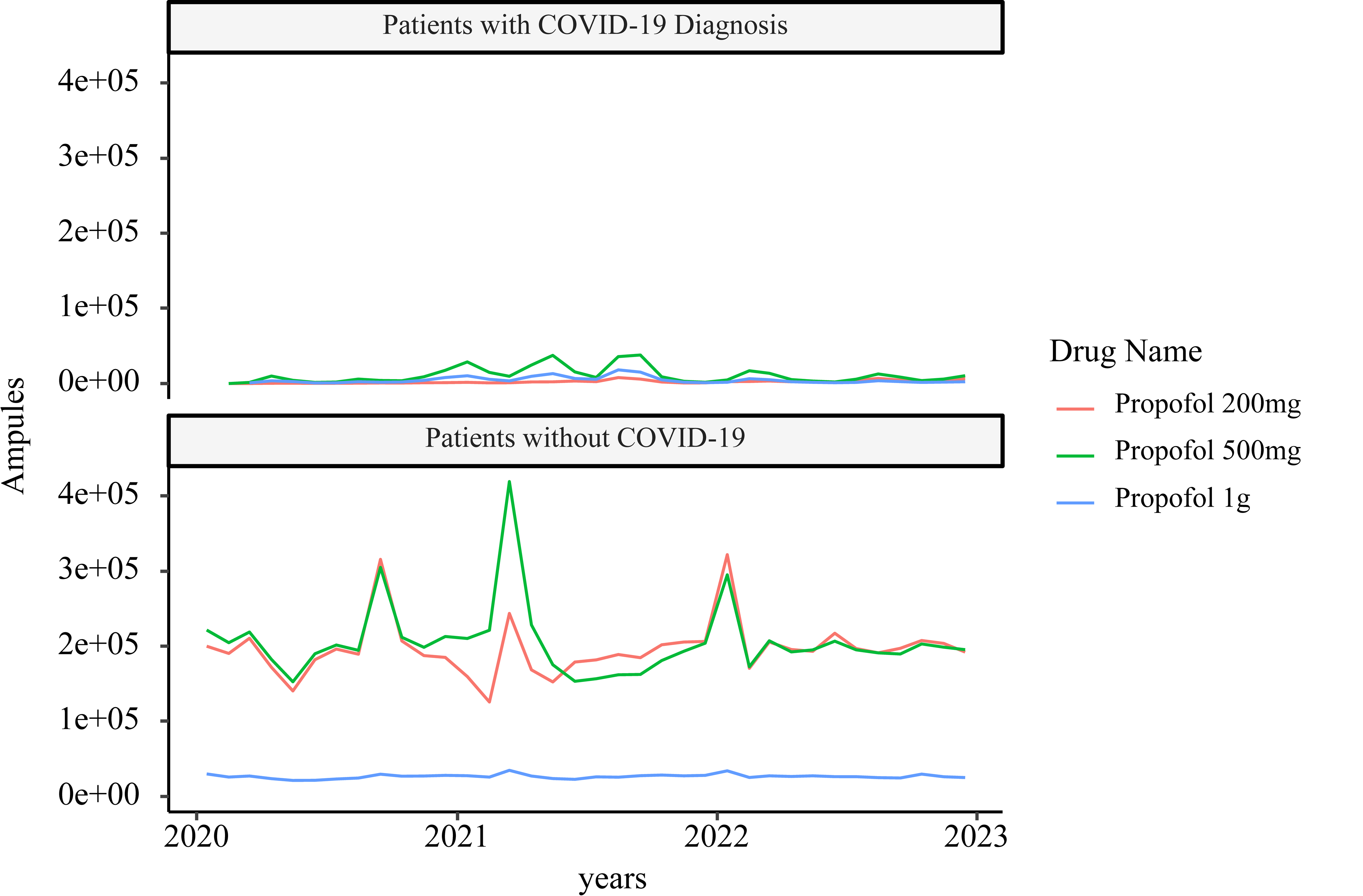

Supplement: S9 Fig — Usage status of propofol over time, stratified by the presence or absence of COVID-19 infection. (TIF) [file pone.0303493.s009.tif]

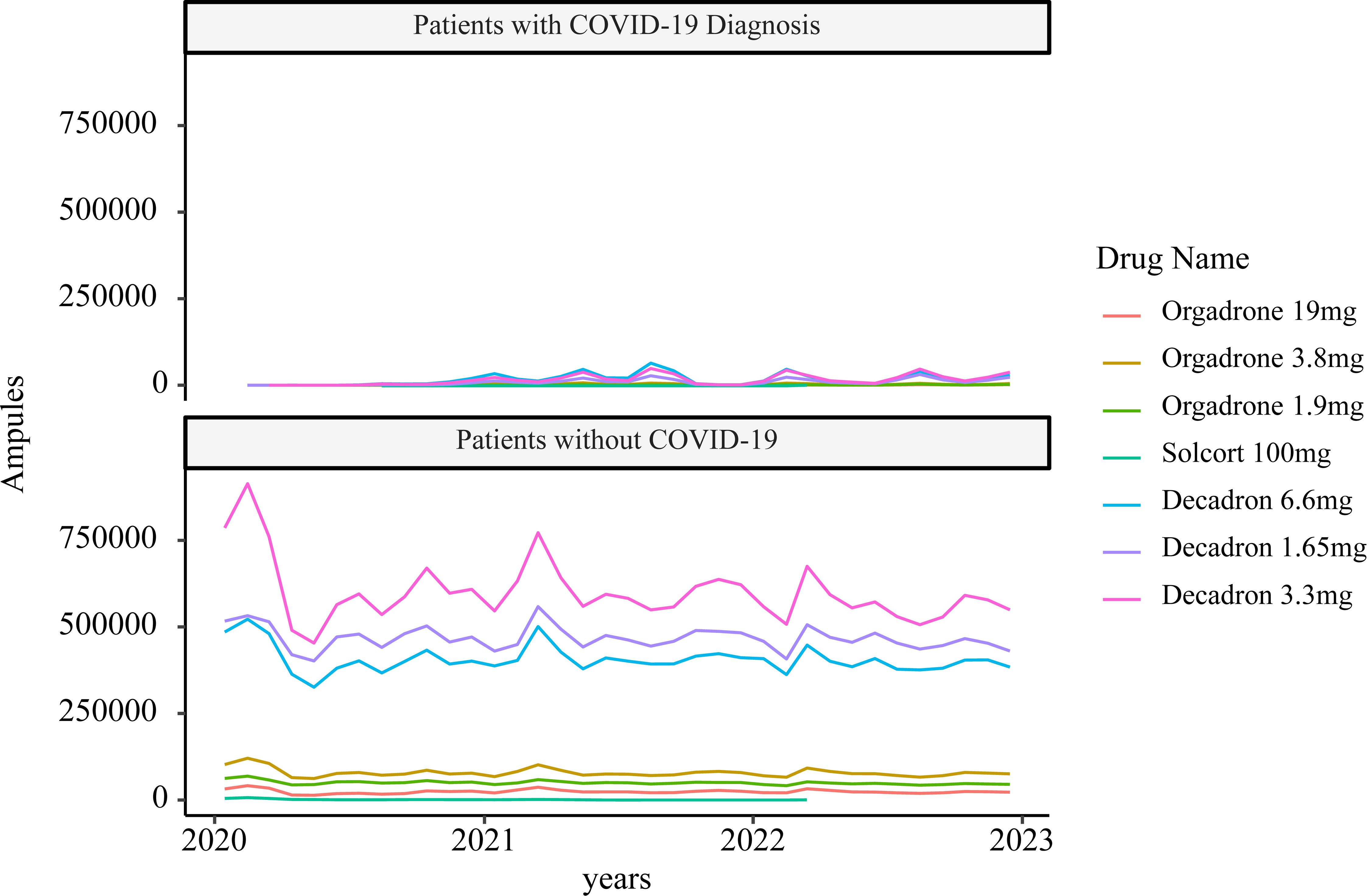

Supplement: S10 Fig — Usage status of corticosteroid injection over time, stratified by the presence or absence of COVID-19 infection. (TIF) [file pone.0303493.s010.tif]

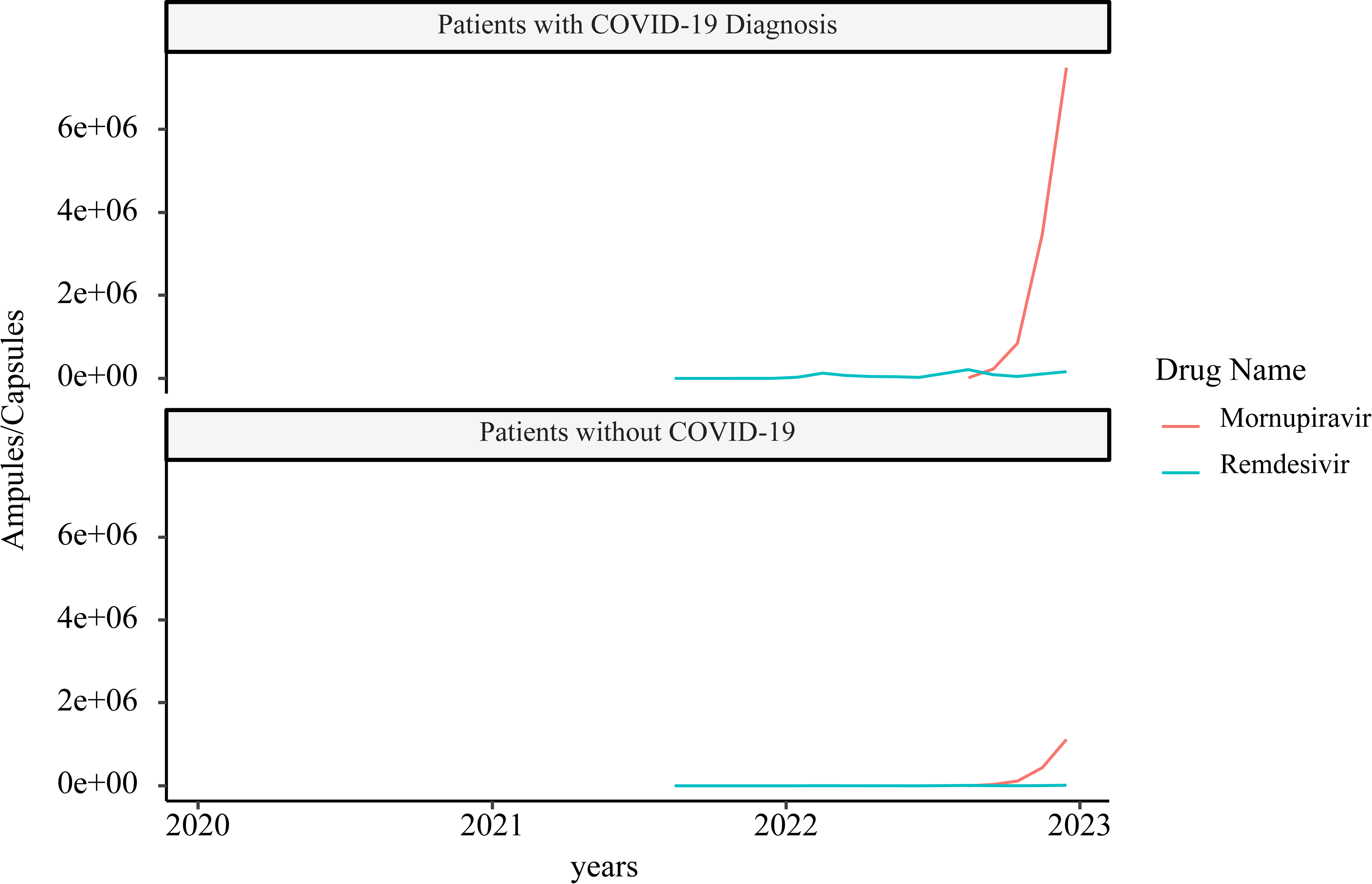

Supplement: S11 Fig — Usage status of antiviral agents for COVID-19 over time, stratified by the presence or absence of COVID-19 infection. (TIF) [file pone.0303493.s011.tif]

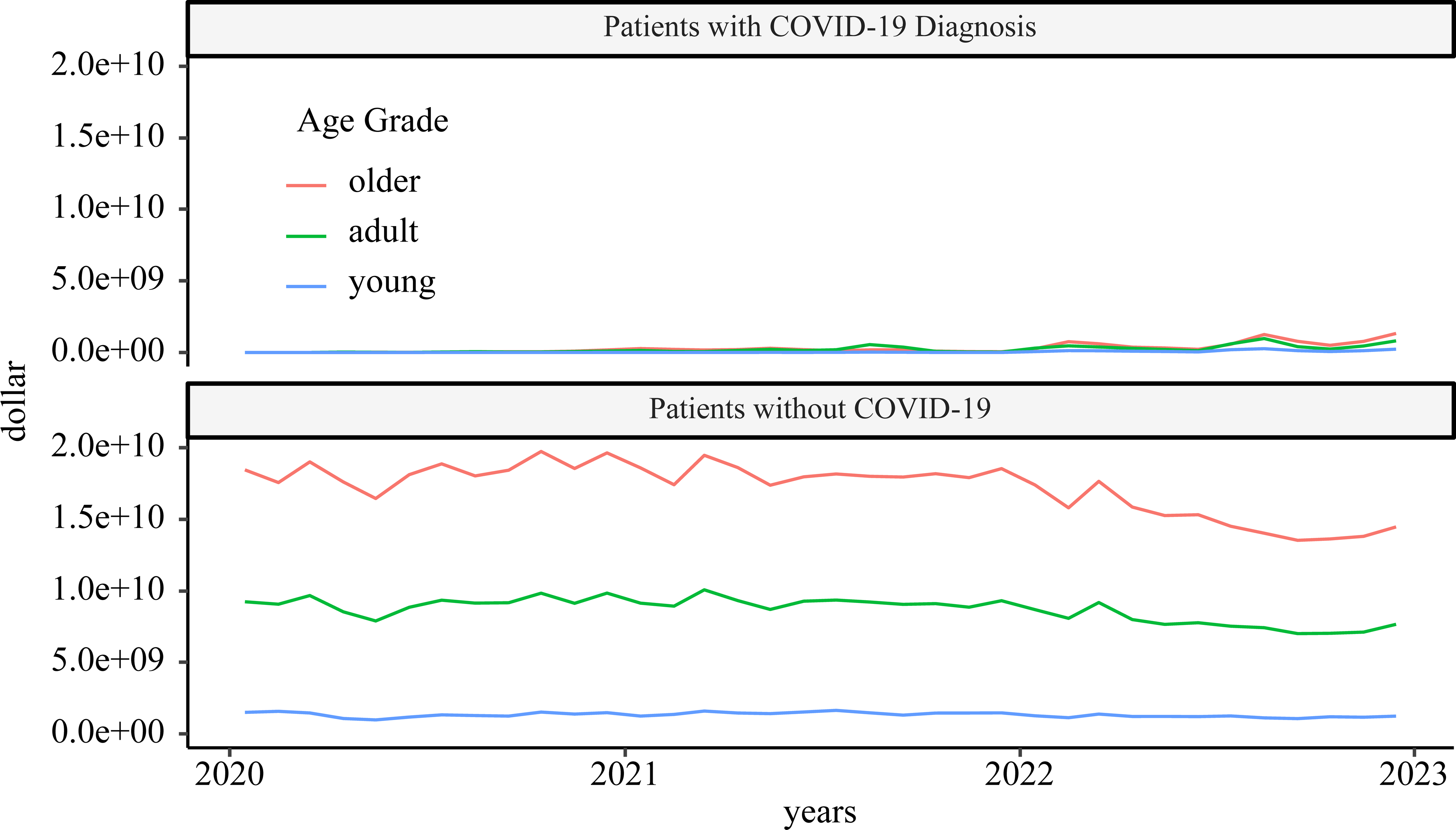

Supplement: S12 Fig — The red arrow indicates the 5th wave. During this wave, the percentage of medical care invested in patients with COVID-19 was smaller than that in those without COVID-19. Although medical expenses appeared to have declined, such decline was caused by the impact of the yen’s depreciation. (TIF) [file pone.0303493.s012.tif]
