# Supplementary material for: Medical resource usage for COVID-19 evaluated using the National Database of Health Insurance Claims and Specific Health Checkups of Japan
Source: PLoS One. 2024 May 13;19(5):e0303493. doi: 10.1371/journal.pone.0303493 (PMC11090316; doi:10.1371/journal.pone.0303493)
Supplement: S1 File — (DOCX) [file pone.0303493.s015.docx]

Medical resource usage for COVID-19 evaluated using the National Database of Health Insurance

Claims and Specific Health Checkups of Japan

Technical detail about the method used in this study

Keita Fukuyama

February 13, 2024

# Introduction

This document describes the details of the receipt data analysis in the present study. The analysis was performed in the following steps:

1. Local DB construction
   1. Local database creation
   2. Bulk inserting
2. Analysis of local DB
   1. Total patient distribution information
   2. Total number of infected people in each medical treatment month
   3. Aggregation of bed usage status and respiratory support
   4. Aggregation of drug usage
   5. Aggregation of viral test
   6. Aggregation of insurance claims
   7. Aggregation of other respiratory viral infections

The details of each step are described below.

In the present research work, publishing the dataset that were used in the research was not permitted because of the rules for using NDB based on paragraph (2) of Article 16 of the Act on Assurance of Medical Care for Elderly People in Japan. The codes, queries, and output content of processing are published on GitHub within the permitted files (https://github.com/fk506cni/ndb_covid_infection)

This document does not describe the relationship between the contents of clinical practice in Japan’s insurance medical care and the receipt records generated at that time.

The code masters for medicines, tests, medical procedures are obtained from the payment fund website(<https://www.ssk.or.jp/seikyushiharai/tensuhyo/index.html>).

# Abbreviations used in the NDB analysis

- NDB: the National Database of Health Insurance Claims and Specific Health Checkups of Japan
- DPC: Diagnosis Procedure Combination (receipt)
- MED: Medical (receipt)
- PHA: Pharmacy (receipt)
- RE: Receipt
- CD: Coding data
- IY: Iyakuhin (drug)
- SI: Sinryou koui (medical procedure)
- SB: Shoubyou (disease code)
- SY: Shoubyoumei (disease name code)
- HO: Hokensha (insurer)
- KO: Kouhi (public expense burden)
- CZ: Chouzai jouhou (dispensing information)
- HER-SYS: Information acquisition and management system for new COVID-19 cases provided by the Ministry of Health, Labor, and Welfare

(https://covid19.mhlw.go.jp/extensions/public/index.html)

# Detail

1. Local database creation

We extracted the data necessary for this research from the NDB created in Amazon Redshift and bulk inserted it into the PostgreSQL DB created on the local drive of the analysis environment. At this time, column selection, filtering, stacking, duplicate flag creation, and so on were performed for each table.

The receipt types and tables on the NDB that were subjected to extraction are described below.

A local database was constructed using the practical month as the partition key.

1. DPC (RE, CD, IY, SI, SB, SY, HO, KO)
2. MED (RE, IY, SI, SY, HO, KO)
3. PHA (RE, IY, CZ, HO, KO)

For pharmaceuticals (IY) and medical services (SI), the table on the NDB side had a horizontal style with the column showing date information. The IY and SI tables in DPC were changed to a vertical format for subsequent analyses.

For payment information (HO and KO), we merged the receipt categories of RE information, checked the public expense burden ratio, and designed a single table in the form of ID1n and claim points for each month.

2. Bulk inserting

We extracted data from the NDB for the DB designed above.

Using an SQL query with a press folder from the DBI package, the necessary data for each treatment month were loaded into chunks according to the NDB partitions and bulk inserted into the local folder.

The queries and inserts are made by month of treatment and table. By parallelizing these processes, the speed is increased. The bulk insert were completed in approximately one week.

Regarding the names of injuries and diseases, only the disease names focused on in this study were extracted.

Moreover, for pharmaceuticals and medical practices, only the codes focused on in this study were extracted.

The CD records in the DPC included records that overlap with SI, IY, and TO (not used this time), and if they were aggregated as it is, the usage fees for medical resources was overestimated.

Therefore, we applied the following duplicate confirmation flag assignment algorithm before the bulk insert.

The algorithm validity was confirmed by the Ministry of Health, Labor, and Welfare and the NDB operating company.

Duplication flag creation algorithm

When there is m of records such as seq2 no: A, date of usage: B, code of drug or procedure: C, dose, or amount: D, usage times: E, practical identity: F in CD, and there is n of records such as seq2 no: A, date of usage: B, code of drug or procedure: C, dose, or amount: D, usage times: E, practical identity: F in IY or SI, we consider which min (m, n) for records in CD is duplicated.

Along with DB construction, the corresponding GitHub folder is described as follows:

- Ps001_create_schema
- Ps002_2_create_ho
- Ps002_3_create_dpccd
- Ps002_4_create_dpcsk
- Ps002_5_create_iy_long
- Ps002_6_create_dpcsi_long
- Ps002_create_whole_db

3. Total patient distribution information

ID1N was used as the individual patient ID in this study.

These are listed along with the age group in the RE records of DPC, MED, and PHA receipts.

Disease name information was listed in the DPC SY, DPC SB, and MED SY tables.

It was output to local storage along with the presence or absence of a COVID-19 diagnosis for each medical treatment month. Furthermore, they were read in chronological order, and the age at the time of first appearance and whether or not there was a COVID-19 infection are determined based on the count value.

The corresponding GitHub folder is Ps003 covid pts dist.

The corresponding Rmd is shown below.
Ps003_S3_id1n_dist

Ps003_S4_id1n_dist_wholeinone

4. Total number of infected people in each medical treatment month
Similar to the above tabulation, the results were grouped by age and tabulated based on the presence or absence of COVID-19 diagnosis for each medical treatment month.

At this time, because it is not necessary to determine the first appearance and count the number of infections, only the aggregated count value is obtained for each month of treatment.

We prepared an SQL query with the treatment month as a placeholder and executed it sequentially to obtain the distribution for each treatment month.

Subsequently, R was used to integrate the results of HER-SYS and visualize the aggregated results.

The corresponding Rmd is shown below.

Ps003_S5_id1n_dist_whole2postgres

Ps003_S6_id1n_dist_postgres2parse

1. Aggregation of bed usage status and respiratory support
   All of these data are listed in the SI and CD tables.

In DPC, the duplication of records in the CD tables is a problem; thus, after filtering the use of the flag created in the previous step, for each ID, calculate how many codes were used in the treatment month.

The usage amount was then aggregated by age group and target code.
The corresponding folder is shown below.
Ps004_cvd_adm_dist

1. Aggregation of drug usage

We calculated the drug charges used in each receipt from DPC:IY+CD, MED:IY, and PHA:IY+CZ and integrated them with the RE table to determine which patients used which drugs and how much.

Additionally, the results were integrated with the RE table, and infection status, age, and usage fees were aggregated by age group, infection status, and drug code.

The results were further aggregated according to drug classification using their ingredients and dose information, and R was used to visualize the changes in usage over time. The corresponding folder is as follows:
Ps005_drug_usage

1. Aggregation of viral test

We calculated the test code used in each receipt from DPC:SI+CD and MED:SI and integrated it with the RE table to determine which patients used which test and how much.

Additionally, we will integrate the RE table with the infection status and age and calculate the usage fees based on age group, infection status, and test code.

The results were further categorized into antibody and antigen tests using test codes and then aggregated. R was used to visualize the changes in usage over time. The corresponding folder is as follows:
Ps06_assay_dist

1. Aggregation of insurance claim

Billing costs for DPC, MED, and PHA were created in advance as an integrated table.

Data will be tabulated by infection status and age group. The corresponding folder is described below.
Ps002_hoko_dist

1. Aggregation of other respiratory viral infections

The same calculations performed to determine the number of infected people in each medical treatment month will also be conducted for influenza virus and RSV infection codes.

The results obtained together with those for COVID-19 were visualized. The corresponding folder is shown below.
Ps07_extra_dis_dist
